# Transport Simulation of Graphene Devices with a Generic Potential in the Presence of an Orthogonal Magnetic Field

**DOI:** 10.3390/nano12071087

**Published:** 2022-03-26

**Authors:** Paolo Marconcini, Massimo Macucci

**Affiliations:** Dipartimento di Ingegneria dell’Informazione, Università di Pisa, Via G. Caruso 16, 56122 Pisa, Italy; m.macucci@mercurio.iet.unipi.it

**Keywords:** graphene device, magnetic field, simulation, envelope function

## Abstract

The effect of an orthogonal magnetic field is introduced into a numerical simulator, based on the solution of the Dirac equation in the reciprocal space, for the study of transport in graphene devices consisting of armchair ribbons with a generic potential. Different approaches are proposed to reach this aim. Their efficiency and range of applicability are compared, with particular focus on the requirements in terms of model setup and on the possible numerical issues that may arise. Then, the extended code is successfully validated, simulating several interesting magnetic-related phenomena in graphene devices, including magnetic-field-induced energy-gap modulation, coherent electron focusing, and Aharonov–Bohm interference effects.

## 1. Introduction

Graphene is a two-dimensional material with very appealing properties, such as high electrical mobility, thermal conductance, and mechanical strength [1,2,3,4]. Moreover, since, as a consequence of its lattice structure, its envelope function equation [5,6,7] coincides with the Dirac–Weyl equation from relativistic quantum mechanics, it represents an ideal testbed for the observation of relativistic-like phenomena at non-relativistic speeds [2,8,9,10,11]. Graphene exhibits also uncommon noise characteristics [12,13,14,15,16]. As a consequence, in the last two decades, a large amount of research has developed on this material [17,18], which has then extended also to other two-dimensional materials [19,20,21].

Up to now, the absence of an energy gap in unconfined pristine monolayer graphene has prevented its usage in digital electronics, even though several approaches have been suggested to overcome this issue by opening a band gap [22,23,24,25,26]. However, several other electronic applications of graphene, above all for the fabrication of sensors, analog devices, and interconnections, have been proposed and are finding their way into industrial products, exploiting its high mobility, atomic thickness (and thus high control of the conduction channel), high surface area to volume ratio, transparency, and flexibility [17,27,28,29]. The atomic thickness of graphene makes it easy to properly shape and modify the device configuration through the electrostatic action of biased gates, which can change the local *n* or *p* nature of graphene [30].

In the presence of a magnetic field, graphene exhibits a very peculiar behavior. For example, in the quantum Hall regime, the Landau levels are not evenly spaced and include a zero-energy level equally shared by electrons and holes [2,10,31,32,33,34,35,36,37]; moreover, snake-states can form and propagate along a *p*–*n* junction [9,38].

From a numerical point of view, the simulation of graphene devices in the presence of an orthogonal magnetic field has been in general performed using a tight-binding description with Peierls substitution [39,40,41]. Due to its atomistic resolution, this approach is particularly suited for the simulation of small-scale devices, while, for large structures, a continuum, envelope-function-based model [9,22,42] is more convenient. However, in the literature, envelope-function-based simulations have usually been performed on basic potential profiles and graphene geometries (often unconfined or with ideal boundary conditions at the edges), or exploiting analytical solutions [43].

Here, we will describe how we have included the spin-independent effect of an orthogonal magnetic field into a code [44] based on the solution in the reciprocal space of the envelope-function equation and on a scattering-matrix approach, which we have previously developed and successfully applied to transport simulations for graphene devices with generic potential landscapes [13,26,45,46]. We have pursued approaches differing in the form of the magnetic vector potential (magnetic gauge) and for the adopted numerical procedures. We will describe them, comparing their performance and discussing the subtle issues that have sometimes to be taken into consideration. We will also show that, in some particularly challenging scenarios, the most performing gauge choice suffers from issues of ill-conditioning, and thus, an alternative gauge has to be adopted.

In order to test the code, we will use it to simulate some known effects of the magnetic field on graphene-based devices: energy-gap modulation, coherent electron focusing, and Aharonov–Bohm conductance oscillations.

The paper is organized in the following way. In Section 2, we will describe the numerical code and the way in which the effect of the magnetic field is introduced, comparing the different approaches we have considered. In Section 3, we will report the results of our simulations for the three cases on which we have chosen to test our code. In Section 4, we will report our conclusions.

## 2. Numerical Method

### 2.1. Graphene Lattice and Ribbon Geometry

Monolayer graphene consists of a hexagonal lattice of $sp^{2}$-hybridized carbon atoms [1]. The graphene lattice constant is $a=\sqrt{3}\;a_{C-C}$, with $a_{C-C}≃0.142$ nm being the distance between nearest-neighbor carbon atoms. The unit cell of the lattice contains two inequivalent carbon atoms, which are commonly indicated with the letters *A* and *B*. The set of all the *A* (*B*) atoms represents the *A* (*B*) sublattice of graphene.

The low-energy transport behavior of graphene is dominated by the $2p_{z}$ atomic orbitals φ of the carbon atoms. In the absence of confinement, they give rise to a conduction and a valence band that touch at two inequivalent degeneration points of the reciprocal space (Dirac points) $\vec{K}$ and $\vec{K^{\prime }}$.

Following a LCAO (linear combination of atomic orbitals) approach, at low energies, the electron wavefunction $\psi (\vec{r})$ at the generic point $\vec{r}$ can be written as:

(1) $$\psi \left(\vec{r}\right)=\sum_{{\vec{R}}_{A}}\psi_{A}\left({\vec{R}}_{A}\right)\varphi \left(\vec{r}-{\vec{R}}_{A}\right)+\sum_{{\vec{R}}_{B}}i\;\psi_{B}\left({\vec{R}}_{B}\right)\varphi \left(\vec{r}-{\vec{R}}_{B}\right)\;,$$

where ${\vec{R}}_{A}$ (${\vec{R}}_{B}$) represents the position of the generic *A* (*B*) carbon atom.

Here, we simulate the transport behavior of a generic armchair graphene ribbon containing $N_{D}$ dimer lines of carbon atoms along its cross-section. Its width (not including the atoms, usually hydrogen, that passivate the edges) is equal to $W=(N_{D}-1)a/2$. We define a reference frame x,y,z, with *x* and *y* the longitudinal (transport) and transverse directions (respectively) on the graphene plane and *z* the direction orthogonal to the graphene plane ($\hat{x},\hat{y},\hat{z}$ are the corresponding unit vectors). Using this reference frame, the Dirac points can be expressed as $\vec{K}=-K\hat{y}$ and $\vec{K^{\prime }}=K\hat{y}$, with K=4π/(3a).

### 2.2. Formulation of the $\vec{k}\cdot \vec{p}$ Problem

If the external potential energy $U(\vec{r})$ varies slowly on the scale of the lattice spacing, for low injection energies, a continuous $\vec{k}\cdot \vec{p}$ description can be formulated to describe transport in graphene [5,6,7]. The components $\psi_{A}\left(\vec{r}\right)$ and $\psi_{B}\left(\vec{r}\right)$ of the electron wave function on the *A* and *B* sublattices, obtained extending by continuity the coefficients $\psi_{A}\left({\vec{R}}_{A}\right)$ and $\psi_{B}\left({\vec{R}}_{B}\right)$ to the generic position $\vec{r}$ of the graphene plane, can be expressed in terms of four envelope functions $F_{\beta }^{\vec{\alpha }}$ (corresponding to the two Dirac points $\vec{\alpha }=\vec{K},\vec{K^{\prime }}$ and to the two sublattices β=A,B) in the following way [7]:

(2) $$\begin{array}{c} \psi_{\beta }\left(\vec{r}\right)=e^{i\vec{K}\cdot \vec{r}}F_{\beta }^{\vec{K}}\left(\vec{r}\right)-i\;e^{i{\vec{K}}^{\prime }\cdot \vec{r}}F_{\beta }^{{\vec{K}}^{\prime }}\left(\vec{r}\right)\;, \end{array}$$

for β=A,B. In a first-order approximation, in monolayer graphene, these envelope functions have to satisfy the following equation (the Dirac–Weyl equation [7]):

(3) $$\left\{\begin{array}{c} \left(v_{F}\hbar \left(\sigma_{x}{\hat{\kappa }}_{x}+\sigma_{y}{\hat{\kappa }}_{y}\right)+U\left(\vec{r}\right)I\right){\vec{F}}^{\vec{K}}=E{\vec{F}}^{\vec{K}}\\ \left(v_{F}\hbar \left(\sigma_{x}{\hat{\kappa }}_{x}-\sigma_{y}{\hat{\kappa }}_{y}\right)+U\left(\vec{r}\right)I\right){\vec{F}}^{\vec{K^{\prime }}}=E{\vec{F}}^{\vec{K^{\prime }}}\;, \end{array}\right.$$

or equivalently

(4) $$\left\{\begin{array}{c} \left(\sigma_{x}{\hat{\kappa }}_{x}+\sigma_{y}{\hat{\kappa }}_{y}+If\left(\vec{r}\right)\right){\vec{F}}^{\vec{K}}=0\\ \left(\sigma_{x}{\hat{\kappa }}_{x}-\sigma_{y}{\hat{\kappa }}_{y}+If\left(\vec{r}\right)\right){\vec{F}}^{\vec{K^{\prime }}}=0\;. \end{array}\right.$$

Here, $\sigma_{x}$ and $\sigma_{y}$ are the Pauli matrices, *I* is the 2×2 identity matrix, $v_{F}$ is the Fermi velocity of graphene, *ℏ* is the reduced Planck constant, *E* is the electron energy, $f\left(\vec{r}\right)=\left(U\left(\vec{r}\right)-E\right)/\left(\hbar v_{F}\right))$, and ${\hat{\kappa }}_{x}$ and ${\hat{\kappa }}_{y}$ are the components of the operator vector $\vec{\hat{\kappa }}$. In the general case in which a magnetic field $\vec{B}$ is present, its effect can be included through a vector potential $\vec{A}$ such that $\vec{B}=\vec{\nabla }\times \vec{A}$. In this case, $\vec{\hat{\kappa }}=-i\vec{\nabla }+\left(e/\hbar \right)\vec{A}$, i.e., ${\hat{\kappa }}_{x}=-i\partial_{x}+\left(e/\hbar \right)A_{x}$ and ${\hat{\kappa }}_{y}=-i\partial_{y}+\left(e/\hbar \right)A_{y}$ (*e* is the elementary charge, while $\partial_{x}$ and $\partial_{y}$ are shorthand forms for ∂/∂x and ∂/∂y, respectively).

The effect of the lateral confinement can be exactly reproduced enforcing Dirichlet boundary conditions at the first points of the two-dimensional hexagonal lattice that fall outside the ribbon, i.e., along the lines of lattice points located a/2 away from the edges of the ribbon [22,23]. Therefore, in a continuum description, the effective width of the ribbon is $\tilde{W}=W+2\left(a/2\right)=\left(N_{D}+1\right)a/2$, and at the effective edges, identified by y=0 and $y=\tilde{W}$ and formed by both *A* and *B* lattice points, we have to enforce the vanishing of both $\psi_{A}$ and $\psi_{B}$:

(5) $$\psi_{\beta }\left(x,y=0\right)=\psi_{\beta }\left(x,y=\tilde{W}\right)=0$$

for β=A,B.

### 2.3. Partition of the Graphene Structure into Slices and Solution within Each Slice

**Figure 1:**
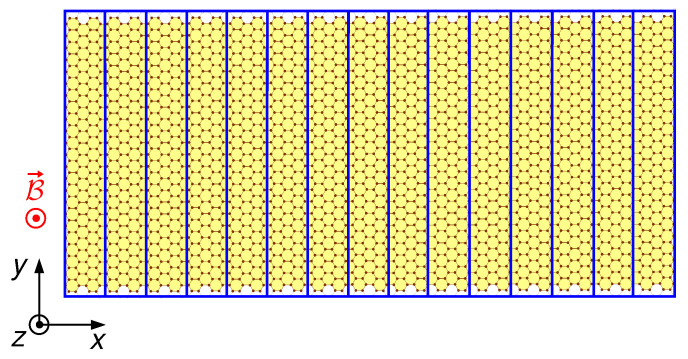
Subdivision of a graphene ribbon into slices.

For the evaluation of the transmission matrix, we use a scattering-matrix approach similar to the method we have previously adopted for the simulation of GaAs/AlGaAs devices [47]. We partition the ribbon into a collection of slices, each one with a width equal to that of the ribbon and a length along the transport direction *x* depending on the chosen solution scheme, so that a particle injected from the entrance lead has to pass through all the slices to reach the exit lead (see Figure 1). We choose the length of the slices in such a way that, within each of them, the lattice geometry does not change and both the potential energy *U* and the vector potential $\vec{A}$ are approximately independent of the transport coordinate *x*. As a consequence, within each slice, the wave function, and thus the envelope functions, can be written (because of translational invariance) as the product of a plane wave propagating in the *x* direction and of a component depending only on *y*:

(6) $$F_{\beta }^{\vec{\alpha }}\left(\vec{r}\right)=e^{i\kappa_{x}x}\Phi_{\beta }^{\vec{\alpha }}\left(y\right)$$

for $\vec{\alpha }=\vec{K},\vec{K^{\prime }}$ and β=A,B.

Exploiting these expressions and defining: $\bar{x}$ as the value of *x* at the center of the considered slice,

(7) $${\vec{\varphi }}^{\vec{K}}\left(y\right)\;=\;\left[\begin{array}{c} \Phi_{A}^{\vec{K}}\left(y\right)\\ \Phi_{B}^{\vec{K}}\left(y\right) \end{array}\right],\;{\vec{\varphi }}^{{\vec{K}}^{\prime }}\left(y\right)\;=\;\left[\begin{array}{c} i\Phi_{A}^{{\vec{K}}^{\prime }}\left(y\right)\\ i\Phi_{B}^{{\vec{K}}^{\prime }}\left(y\right) \end{array}\right],$$

within the slice, Equation (4) becomes

(8) $$\left\{\begin{array}{c} (\sigma_{x}\kappa_{x}+\sigma_{x}\frac{e}{\hbar }A_{x}\left(\bar{x},y\right)-\sigma_{y}i\partial_{y}+\sigma_{y}\frac{e}{\hbar }A_{y}\left(\bar{x},y\right)+\\ \;+If\left(\bar{x},y\right))\;{\vec{\varphi }}^{\vec{K}}\left(y\right)=0\\ (\sigma_{x}\kappa_{x}+\sigma_{x}\frac{e}{\hbar }A_{x}\left(\bar{x},y\right)+\sigma_{y}i\partial_{y}-\sigma_{y}\frac{e}{\hbar }A_{y}\left(\bar{x},y\right)+\\ \;+If\left(\bar{x},y\right))\;{\vec{\varphi }}^{\vec{K^{\prime }}}\left(y\right)=0\;, \end{array}\right.$$

while the boundary conditions (5) become

(9) $${\vec{\varphi }}^{\vec{K}}\left(0\right)={\vec{\varphi }}^{{\vec{K}}^{\prime }}\left(0\right)\;,\;\;{\vec{\varphi }}^{\vec{K}}\left(\tilde{W}\right)=e^{i2K\tilde{W}}{\vec{\varphi }}^{{\vec{K}}^{\prime }}\left(\tilde{W}\right)\;.$$

Premultiplying the two equations of Equation (8) by $-\sigma_{x}$ and reorganizing them, we can write the differential problem as:

(10) $$\left\{\begin{array}{c} -(\sigma_{z}\partial_{y}+I\frac{e}{\hbar }A_{x}\left(\bar{x},y\right)+\sigma_{z}i\frac{e}{\hbar }A_{y}\left(\bar{x},y\right)+\\ \;+\sigma_{x}f\left(\bar{x},y\right))\;{\vec{\varphi }}^{\vec{K}}\left(y\right)=\kappa_{x}{\vec{\varphi }}^{\vec{K}}\left(y\right)\\ -(-\sigma_{z}\partial_{y}+I\frac{e}{\hbar }A_{x}\left(\bar{x},y\right)-\sigma_{z}i\frac{e}{\hbar }A_{y}\left(\bar{x},y\right)+\\ \;+\sigma_{x}f\left(\bar{x},y\right))\;{\vec{\varphi }}^{{\vec{K}}^{\prime }}\left(y\right)=\kappa_{x}{\vec{\varphi }}^{{\vec{K}}^{\prime }}\left(y\right)\\ {\vec{\varphi }}^{\vec{K}}\left(0\right)={\vec{\varphi }}^{{\vec{K}}^{\prime }}\left(0\right)\\ {\vec{\varphi }}^{\vec{K}}\left(\tilde{W}\right)=e^{i2\tilde{K}\tilde{W}}{\vec{\varphi }}^{{\vec{K}}^{\prime }}\left(\tilde{W}\right)\;, \end{array}\right.$$

where we have replaced *K* with the smallest number $\tilde{K}$ such that $e^{i2\tilde{K}\tilde{W}}=e^{i2K\tilde{W}}$, i.e., with $\tilde{K}=K-n_{0}\;\left(\pi /\tilde{W}\right)$, where $n_{0}=\mathrm{round}\left(K\tilde{W}/\pi \right)$.

The solution of this system in real space with standard discretization methods would lead to many spurious solutions as a consequence of the fermion doubling problem [48]. This issue could be avoided by performing a real space solution with a Stacey discretization scheme [44,49,50]; however, a large number of discretization points would be required to obtain results with a sufficient numerical precision [44]. Instead, a much more efficient solution can be obtained recasting the problem into an equivalent one with periodic boundary conditions and performing the solution in the reciprocal space [44,46].

In detail, let us define over the domain $\left[0,2\tilde{W}\right]$ the following two-component function:

(11) $$\vec{\rho }\left(y\right)\;=\;\left\{\begin{array}{cc} \;\;e^{-i\tilde{K}y}\;{\vec{\varphi }}^{\vec{K}}\left(y\right) & \;y\;\in \;[0,\tilde{W}]\\ \;\;e^{i\tilde{K}\left(2\tilde{W}-y\right)}\;{\vec{\varphi }}^{{\vec{K}}^{\prime }}\left(2\tilde{W}-y\right) & \;y\;\in \;[\tilde{W},2\tilde{W}]\;. \end{array}\right.$$

Writing the first equation of Equation (10) in terms of $\vec{\rho }\left(y\right)$, for $y\;\in \;[0,\tilde{W}]$, we obtain:

(12) $$\begin{array}{cc} \; & -(\sigma_{z}\partial_{y}+\sigma_{z}i\tilde{K}+I\frac{e}{\hbar }A_{x}\left(\bar{x},y\right)+\sigma_{z}i\frac{e}{\hbar }A_{y}\left(\bar{x},y\right)+\\ \; & \;+\sigma_{x}f\left(\bar{x},y\right))\vec{\rho }\left(y\right)=\kappa_{x}\vec{\rho }\left(y\right)\;, \end{array}$$

while replacing *y* with $2\tilde{W}-y$ in the second equation of Equation (10) and writing it in terms of $\vec{\rho }\left(y\right)$, for $y\;\in \;[\tilde{W},2\tilde{W}]$, we have:

(13) $$\begin{array}{cc} \; & -(\sigma_{z}\partial_{y}+\sigma_{z}i\tilde{K}+I\frac{e}{\hbar }A_{x}\left(\bar{x},2\tilde{W}-y\right)-\\ \; & \;-\sigma_{z}i\frac{e}{\hbar }A_{y}\left(\bar{x},2\tilde{W}-y\right)+\sigma_{x}f\left(\bar{x},2\tilde{W}-y\right))\vec{\rho }\left(y\right)=\\ \; & \;=\kappa_{x}\vec{\rho }\left(y\right)\;. \end{array}$$

Moreover, in terms of $\vec{\rho }\left(y\right)$, the third equation of Equation (10) becomes $\vec{\rho }\left(2\tilde{W}\right)=\vec{\rho }\left(0\right)$, while the fourth one represents a continuity condition on $\vec{\rho }\left(y\right)$ in $y=\tilde{W}$.

Therefore, Equation (10) is equivalent to the following system over the domain $\left[0,2\tilde{W}\right]$:

(14) $$\left\{\begin{array}{c} \;\;-\;\;\left(\;\sigma_{z}\partial_{y}\;+\;\sigma_{z}i\tilde{K}\;+\;Ib\left(y\right)\;+\;\sigma_{z}c\left(y\right)\;+\;\sigma_{x}h\left(y\right)\;\right)\;\vec{\rho }\left(y\right)\;=\;\kappa_{x}\vec{\rho }\left(y\right)\\ \;\vec{\rho }\left(2\tilde{W}\right)\;=\;\vec{\rho }\left(0\right)\;, \end{array}\right.$$

with

(15) $$\begin{array}{cc} \; & b\left(y\right)=\frac{e}{\hbar }A_{x}(\bar{x},\tilde{W}-|\tilde{W}-y|)),\\ \; & c\left(y\right)=i\frac{e}{\hbar }\mathrm{sign}\left(\tilde{W}-y\right)A_{y}(\bar{x},\tilde{W}-|\tilde{W}-y\left|\right),\\ \; & h\left(y\right)=f(\bar{x},\tilde{W}-|\tilde{W}-y|). \end{array}$$

Extending by periodicity, with period $2\tilde{W}$, the functions $\vec{\rho }\left(y\right)$, b(y), c(y), and h(y), Equation (14) can be seen as a differential eigenproblem with eigenvalue $\kappa_{x}$ and periodic boundary conditions on the eigenfunction $\vec{\rho }\left(y\right)$. This can be very efficiently solved working in the reciprocal space in the following way.

In the first of Equation (10), the functions $\vec{\rho }\left(y\right)$, b(y), c(y), and h(y) (extended by periodicity) can be replaced by their Fourier expansions (expressed in terms of the Fourier coefficients ${\vec{a}}_{m}$, $b_{\ell }$, $c_{\ell }$, and $h_{\ell }$, respectively):

(16) $$\begin{array}{c} \vec{\rho }\left(y\right)=\;\;\;\;\sum_{m=-\infty }^{\infty }\;\;{\vec{a}}_{m}\;e^{im\frac{\pi }{\tilde{W}}y}\;,\;b\left(y\right)=\;\;\;\;\sum_{\ell =-\infty }^{\infty }\;\;b_{\ell }\;e^{i\ell \frac{\pi }{\tilde{W}}y}\;,\;\\ c\left(y\right)=\;\;\;\;\sum_{\ell =-\infty }^{\infty }\;\;c_{\ell }\;e^{i\ell \frac{\pi }{\tilde{W}}y}\;,\;h\left(y\right)=\;\;\;\;\sum_{\ell =-\infty }^{\infty }\;\;h_{\ell }\;e^{i\ell \frac{\pi }{\tilde{W}}y}\;. \end{array}$$

The quantities ${\vec{a}}_{m}$’s are the unknowns, while $b_{\ell }$, $c_{\ell }$, and $h_{\ell }$ can be computed from the (known) behavior of the vector and electrostatic potentials by using analytical expressions or optimized FFT (fast Fourier transform) routines. In particular, since b(y) and h(y) are real and even functions, their Fourier coefficients are real, with the property that $b_{-n}=b_{n}$ and $h_{-n}=h_{n}$ for every integer *n*. Instead, since c(y) is an imaginary and odd function, its Fourier coefficients are real, with the property that $c_{-n}=-c_{n}$.

Projecting the resulting equation onto the set of basis functions $e^{i(\pi n/\tilde{W})y}$ (with *n* a generic integer number), we obtain:

(17) $$\begin{array}{cc} \; & -\;\;\sum_{m=-\infty }^{+\infty }\;\;(\sigma_{z}i(\frac{\pi n}{\tilde{W}}+\tilde{K})\delta_{n,m}+Ib_{n-m}+\sigma_{z}c_{n-m}+\\ \; & \;+\sigma_{x}h_{n-m}){\vec{a}}_{m}=\kappa_{x}{\vec{a}}_{n}\;\mathrm{for}\;n=-\infty ,\ldots ,+\infty \end{array}$$

These relations, taken as a whole, represent an infinite-dimensional linear eigenproblem with eigenvalue $\kappa_{x}$ and eigenvector given by the vertical concatenation of the Fourier coefficients ${\vec{a}}_{n}$ (each with two components) of the function $\vec{\rho }\left(y\right)$. The matrix elements (each identified by a row index *n* and a column index *m*) of the linear system are given by the argument of the sum.

The first term of the sum contributes only to the diagonal of the matrix; moreover, due to our choice of $\tilde{K}$, $|\left(\pi n/\tilde{W}\right)+\tilde{K}|$ is minimum for n=0, while it increases as |n| is increased. Instead, if b(y), c(y), and h(y) are square-integrable, their Fourier coefficients $b_{n-m}$, $c_{n-m}$, and $h_{n-m}$ vanish for sufficiently large values of their order |n−m|, i.e., moving sufficiently away from the diagonal of the matrix. For sufficiently large values of |n|, the elements on the diagonal dominate over those outside the diagonal. Therefore, if we define an integer *D* such that

(18) $$\left|\frac{\pi D}{\tilde{W}}+\tilde{K}\right|\gg \max_{j}|b_{j}|,\;\max_{j}|c_{j}|,\;\max_{j}\left|h_{j}\right|\;,$$

the overall matrix can be approximately seen as a block-diagonal matrix with three blocks, containing the following elements: with n<−D and m<−D, with |n|<D and |m|<D, and with n>D and m>D, respectively. The first and the third blocks are nearly diagonal and would yield high-order Fourier coefficients ${\vec{a}}_{n}$ and eigenvalues with a very large imaginary part, which typically give a negligible contribution in transport calculations. Therefore, in our calculation, we consider only the part of the matrix with |n|<D and |m|<D. This actually corresponds to a frequency cut-off: the solutions ${\vec{a}}_{n}$ and $\kappa_{x}$ of this truncated system represent a good approximation of the Fourier coefficients of the slow-varying eigenfunctions of Equation (14) and of their corresponding wave vectors.

From these solutions, we can reconstruct the function $\vec{\rho }\left(y\right)$ by using Equation (16), and thus (from Equation (11)):

(19) $$\begin{array}{cc} \; & {\vec{\varphi }}^{\vec{K}}\left(y\right)=e^{i\tilde{K}y}\sum_{n=-D}^{D}{\vec{a}}_{n}\;e^{in\frac{\pi }{\tilde{W}}y}\;,\\ \; & {\vec{\varphi }}^{{\vec{K}}^{\prime }}\left(y\right)=e^{-i\tilde{K}y}\sum_{n=-D}^{D}{\vec{a}}_{n}\;e^{-in\frac{\pi }{\tilde{W}}y}\;. \end{array}$$

Finally, using Equations (2), (6) and (7), the corresponding wave functions on the two sublattices (β=A,B) can be expressed as (if $a_{nA}$ and $a_{nB}$ are the two components of ${\vec{a}}_{n}$):

(20) $$\begin{array}{cc} \psi_{\beta }\left(x,y\right) & =2\;i\sum_{n=-D}^{D}\left(a_{n\beta }\;\sin\left(\left(n-n_{0}\right)\frac{\pi }{\tilde{W}}y\right)\right)e^{i\kappa_{x}x}\\ \; & =\chi_{\beta }\left(y\right)\;e^{i\kappa_{x}x}\;, \end{array}$$

which represent the eigenmodes of the slice that we are considering. Within this slice, a generic wave function is a linear combination of these modes.

### 2.4. Recursive Scattering-Matrix Transport Solution

Once we have computed the eigenmodes of the different slices of the device, we perform the transport analysis using a recursive scattering-matrix approach.

We consider the device as consisting of the following: a region extending from the entrance (i.e., the beginning of the first slice) to the middle of the second slice, a region extending from the middle of the second slice to the middle of the third slice, and so on for the following ones, up to the last region, which extends from the middle of the second-last slice to the exit (i.e., the end of the last slice). Each region includes only one interface between adjacent slices (a left slice and a right slice) and thus contains, at most, only one discontinuity.

We evaluate the scattering matrix of each region using the following mode-matching technique. Exploiting the linearity of the problem, we inject a single mode at a time into the region. This is done for each of the eigenmodes of the left slice (injecting them in the rightward direction) and for each of the eigenmodes of the right slice (injecting them in the leftward direction).

Each time, at the interface between the two slices, we enforce the continuity of $\psi_{A}\left(x,y\right)$ and $\psi_{B}\left(x,y\right)$, which are expressed as linear combinations of the different modes with coefficients given by the reflection and transmission coefficients of the scattering matrix. For example, assuming to inject the *i*-th mode from the left slice, we enforce the following continuity equations for the two sublattices β=A,B:

(21) $$\begin{array}{cc} \; & \chi_{\beta i}^{l+}\left(y\right)\;e^{i\kappa_{xi}^{l+}\left(x_{int}-x_{in}\right)}+\sum_{n}r_{ni}\chi_{\beta n}^{l-}\left(y\right)\;e^{i\kappa_{xn}^{l-}\left(x_{int}-x_{in}\right)}=\\ \; & \;=\sum_{n}t_{ni}\chi_{\beta n}^{r+}\left(y\right)\;e^{i\kappa_{xn}^{r+}\left(x_{int}-x_{out}\right)}\;, \end{array}$$

where *l* (*r*) refers to eigenmodes of the left (right) slice, + (−) refers to rightgoing (leftgoing) modes, $r_{ni}$ ($t_{ni}$) is the reflection (transmission) coefficient from the *i*-th mode to the *n*-th one, and $x_{in}$, $x_{int}$, and $x_{out}$ are the values of *x* at the entrance of the region, at the interface, and at the exit of the region, respectively.

These continuity equations are projected onto a basis consisting of functions of the form $\sin(\left(j-n_{0}\right)\pi y/\tilde{W})$. In detail, for each injected mode, we perform a spinorial projection of the set of the two continuity equations written for the two sublattices (Equation (21) for β=A,B) onto two-component vectors with both components given by the basis of sines, and performing a single-component projection of the continuity equation written on the *B* sublattice (Equation (21) for β=B) onto the basis of sines [51]. In this way, we obtain a linear system in the reflection and transmission coefficients, yielding the scattering matrix of the region connecting the two slices.

**Figure 2:**
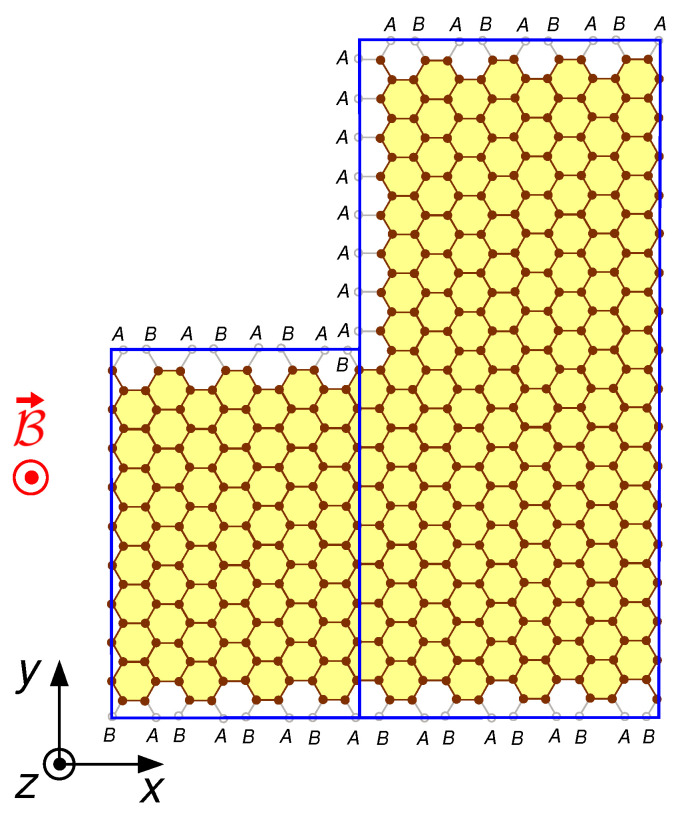
Case with two adjacent slices with edges not aligned; in this case, α=A (as defined in the text).

If the upper and/or lower edges of the two adjacent slices are not aligned, for example if the two slices have different widths, the linear system has to be written using a generalized procedure (see Figure 2). In this case, the interface includes vertical segments of the effective boundaries of the device (generalizing what has been done for the upper and lower edges, the effective boundaries of the device, where the wave function has to vanish, are defined one lattice point outside the structure of carbon atoms [22,23]). Since these vertical segments of the effective boundary contain atoms that belong to a single sublattice (let us define it α, with α=A or *B* depending on the geometry), at such atoms, we have to enforce the vanishing only of $\psi_{\alpha }\left(x,y\right)$. Instead, on the part of the interface that does not include any effective boundary section, we have to enforce the continuity of the wave function on both the *A* and *B* sublattices. This is obtained by performing a spinorial projection of the set of the two continuity equations written for the two sublattices onto two-component vectors with both components given by a basis of sines of the portion of the interface not including effective boundary sections, and performing a single-component projection of the continuity equation written for the α sublattice onto a basis of sines of the overall interface.

All projections are computed operating directly in the Fourier space, without the need to go back to the direct space through an inverse Fourier transform. This allows a further speed up in the calculations.

Once we have computed the scattering matrices of all the regions, we compose them using a standard procedure [52], obtaining the scattering matrix *S*, which relates the amplitudes of the modes at the entrance and at the exit of the overall device (and its transmission matrix *t*, which is just a submatrix of *S*). Finally, we exploit the transmission coefficients $t_{nm}$ of the device (i.e., the elements of the transmission matrix *t*) to compute the conductance of the device using the Landauer–Büttiker formula [52,53,54]:

(22) $$G=\frac{2\;e^{2}}{h}\sum_{n,m}{\left|t_{nm}^{\prime }\right|}^{2}\;.$$

Here, *h* is the Planck constant, and $t_{nm}^{\prime }=t_{nm}\sqrt{|I_{xn}\left|/\right|I_{xm}|}$, where $t_{nm}$ is the transmission coefficient of the device from the *m*-th input mode to the *n*-th output mode, and $I_{xi}$ is the probability current in the *x* direction of the *i*-th mode. The sum is performed only on the modes, specified by the indices *m* and *n*, which propagate with $I_{x}\neq 0$ at the entrance and exit leads, respectively. The presence of a magnetic field affects the envelope functions but does not alter the relationship between the probability current and the envelope functions; therefore, $I_{xi}$ is still given by

(23) $$\begin{array}{c} I_{xi}=v_{F}\int_{0}^{2\tilde{W}}\vec{\rho_{i}}{\left(y\right)}^{†}\sigma_{x}\vec{\rho_{i}}\left(y\right)dy\;. \end{array}$$

The influence of the temperature on the value of the conductance can be included by averaging the result of Equation (22) on the energy *E*, using as a weight the difference between the Fermi–Dirac occupation functions at the two contacts [52]:

(24) $$\tilde{G}=\frac{2e}{hV}\int_{-\infty }^{+\infty }\;\;\sum_{n,m}{\left|t_{nm}^{\prime }\left(E\right)\right|}^{2}\left\{f\left[E-\left(\mu_{0}+eV\right)\right]-f\left[E-\mu_{0}\right]\right\}dE$$

where *V* is the voltage difference between the two contacts, $\mu_{0}$ and $\mu_{0}+eV$ are the electrochemical potentials of the two contacts, and $f\left[E\right]=1/[1+\exp\left(E/K_{B}T\right)]$ is the Fermi–Dirac occupation function (with $K_{B}$ the Boltzmann constant and *T* the absolute temperature).

With this recursive scattering matrix approach, the effect of the dependence on *x* of the factors influencing transport (*U*, $\vec{A}$, ribbon structure) emerges through the scattering action that their variations along *x* cause at the interface between different slices (i.e., emerges only when composing the scattering matrices of the slices). Instead, their dependence on *y* is numerically handled at the level of the single slices (and thus, the effect on transport of this dependence already appears during the numerical treatment of the single slices).

### 2.5. First Choice of the Magnetic Gauge and Possible Ill-Conditioning Problems

As we have seen, in the envelope-function equation, the effect of the magnetic field appears through the vector potential. However, for a given magnetic field $\vec{B}\left(x,y\right)$, infinite vector potentials $\vec{A}\left(x,y\right)$ exist such that $\vec{B}\left(x,y\right)=\vec{\nabla }\times \vec{A}\left(x,y\right)$. In particular, in our simulations, we have considered a constant magnetic field orthogonal to the graphene plane $\vec{B}=B\hat{z}$, and we have adopted the following two alternative choices for the vector potential: $\vec{A}=-By\hat{x}$ and $\vec{A}=Bx\hat{y}$ (the Landau gauges), which are generally the most convenient ones for rectangular geometries [55].

Let us first consider the choice $\vec{A}=-By\hat{x}$ (which in the following will be referred as “first gauge”).

In this case, the vector potential does not depend on *x*, and thus, it does not introduce a constraint for the choice of the slice length, which, therefore, is limited only by the geometry of the device and by the variation along *x* of the electrostatic potential U(x,y).

Inside each slice with longitudinally constant geometry and electrostatic potential, adopting this gauge, we have that (on the domain $\left[0,2\tilde{W}\right]$):

(25) $$\begin{array}{cc} \; & b\left(y\right)=\frac{e}{\hbar }A_{x}(\bar{x},\tilde{W}-|\tilde{W}-y|))=-\frac{e}{\hbar }B\;(\tilde{W}-|\tilde{W}-y\left|\right)\;,\\ \; & c\left(y\right)=i\frac{e}{\hbar }\mathrm{sign}\left(\tilde{W}-y\right)A_{y}(\bar{x},\tilde{W}-|\tilde{W}-y\left|\right)=0\;, \end{array}$$

which are then extended by periodicity with period $2\tilde{W}$. Therefore, b(y) is an even real function given by a triangular wave ranging from 0 (value for y=0 and $y=2\tilde{W}$) to $-\left(e/\hbar \right)B\tilde{W}$ (value for $y=\tilde{W}$), while c(y) is identically null.

The Fourier coefficients of b(y) are analytically known:

(26) $$b_{2k}=\left\{\begin{array}{cc} \;\;-\frac{eB\tilde{W}}{2\hbar } & \mathrm{for}\;k=0\\ \;\;0 & \mathrm{for}\;k\neq 0 \end{array}\right.,\;\;b_{2k+1}=\frac{2eB\tilde{W}}{\hbar {\left(\left(2k+1\right)\pi \right)}^{2}}$$

for all the integer numbers *k*, while all the Fourier coefficients of c(y) are null: $c_{n}=0$ for every integer *n*.

In the absence of a magnetic field, we know [44] that if $\kappa_{x}$ is an eigenvalue of the system (14) also $\kappa_{x}^{*}$, $-\kappa_{x}$ and $-\kappa_{x}^{*}$ are eigenvalues of (14). In the presence of a magnetic field, using this gauge, the fourfold symmetry in the Gauss plane disappears: if $\kappa_{x}$ is an eigenvalue of (14), we can only state that also $\kappa_{x}^{*}$ is an eigenvalue of (14).

The use of this gauge in transport simulations presents several advantages. First of all, as we have already noticed, in this case, the variation of the vector potential does not represent a constraint for the length of the slices, since $\vec{A}$ depends only on *y*. Therefore, if the ribbon structure and the electrostatic potential *U* do not undergo fast variations in the *x* direction, the simulation can be performed dividing the device into a relatively small number of slices. Moreover, in each slice, the function b(y) has Fourier coefficients that decay quite rapidly (quadratically) with their order, thus limiting the size of the eigenvalue problem to be solved. This makes it possible to perform very rapid transport simulations as long as the solution method remains well-conditioned.

However, for high values of the device width $\tilde{W}$ and of the magnetic field B, the choice of this gauge yields severe numerical problems. Indeed, when $B\tilde{W}>200$ T nm, depending on the specific injection energy and potential profile, the eigenproblem may become ill-conditioned (for most of the eigenvalues, the modulus of the inner product between the left and right normalized eigenvectors is much less than one [56]). In these cases, the numerical solution of the eigenproblem yields a large amount of randomly dispersed, non-physical spurious eigenvalues, and this simulation approach fails (in order to exclude algorithm-specific numerical problems, we have also repeated the calculation using routines from different packages: LAPACK [57], ARPACK [58], JDQZ [59], CS [60,61], FEAST [62], obtaining, however, analogous results).

### 2.6. Second Choice of the Magnetic Gauge and Importance of the Choice of Contact Model

The other gauge choice we have considered is $\vec{A}=Bx\hat{y}$ (in the following, it will be referred to as “second gauge”).

In this case, the vector potential $\vec{A}$ depends on *x*; therefore, the length of the slices has to be chosen in such a way that, within each slice, not only the ribbon structure and U(x,y), but also $\vec{A}\left(x,y\right)$ approximately do not change with *x*. This last condition is satisfied choosing the length Δx of each slice is such a way that the flux of the magnetic field through the slice $B\left(\Delta x\right)\tilde{W}=\left(\Delta \right|\vec{A}\left|\right)\tilde{W}$ is much less than the magnetic flux quantum h/e. Under this constraint, the steplike approximation adopted for the vector potential (with $|\vec{A}|$ constant and equal to $B\;\bar{x}$ within each slice, $\bar{x}$ being the coordinate at the center of the slice) has a negligible effect on the calculation.

Using this gauge, within each slice with longitudinally constant geometry, electrostatic potential, and vector potential, we have that

(27) $$\begin{array}{cc} b(y) & =\frac{e}{\hbar }A_{x}(\bar{x},\tilde{W}-|\tilde{W}-y|))=0\;,\\ c(y) & =i\frac{e}{\hbar }\mathrm{sign}\left(\tilde{W}-y\right)A_{y}(\bar{x},\tilde{W}-|\tilde{W}-y\left|\right)=\\ \; & =i\frac{e}{\hbar }\mathrm{sign}\left(\tilde{W}-y\right)B\;\bar{x}\;, \end{array}$$

which are then extended by periodicity with period $2\tilde{W}$. In this case, b(y) is identically null, while c(y) is an odd imaginary function given by a square wave with zero average, equal to $i\left(e/\hbar \right)B\;\bar{x}$ between y=0 and $y=\tilde{W}$, and to $-i\left(e/\hbar \right)B\;\bar{x}$ between $y=\tilde{W}$ and $y=2\tilde{W}$.

All the Fourier coefficients of b(y) are null: $b_{n}=0$ for every integer *n*, while the Fourier coefficients of c(y) are analytically known:

(28) $$c_{2k}=0\;,\;c_{2k+1}=\frac{2eB\;\bar{x}}{\hbar (2k+1)\pi }$$

for all the integer numbers *k*.

Using this gauge, the solutions of (14) in the presence of a magnetic field preserve the fourfold symmetry in the Gauss plane observed in the absence of a magnetic field: if $\kappa_{x}$ is an eigenvalue of (14), also $\kappa_{x}^{*}$, $-\kappa_{x}$ and $-\kappa_{x}^{*}$ are eigenvalues of (14).

The difference, at the level of the single slice, with respect to the previous gauge, is not surprising. Indeed, using the previous gauge (which treats the magnetic field through a vector potential depending only on *y*), the effect of the magnetic field on transport is expressed already within each single slice. Instead, with this “second gauge” (in which the vector potential depends on *x*), the effect of the magnetic field is expressed only taking into account the scattering action by the discontinuities of the vector potential between adjacent slices. However, as it should be, at the end of the calculation, the physically meaningful quantities, such as the conductance of the device, do not depend on the adopted gauge.

Since the dependence of $\vec{A}$ on *x* limits the maximum length of the slices, in general, it is necessary to divide the structure into a larger number of slices. This increases the simulation time with respect to the previous gauge. Moreover, for each slice, in principle, it would be necessary to solve an eigensystem with a larger matrix size, because, in this case, the eigenproblem (14) contains the square-wave function c(y), which, due to its abrupt discontinuities, has Fourier coefficients that decrease slowly (linearly) with their order.

However, this second drawback can be circumvented in the following way. Let us indicate with the subscript “0” the solutions in the absence of a magnetic field, and, in particular, let us define ${\kappa_{x}}_{0}$ as the generic eigenvalue of our eigensystem (14) for B=0 and ${\vec{\rho }}_{0}\left(y\right)$ as the corresponding eigenfunction. It is easy to verify that, using this “second gauge,” the eigensystem (14) for B≠0 has the same eigenvalue $\kappa_{x}={\kappa_{x}}_{0}$ (in agreement with our previous observation that the fourfold symmetry of the eigenvalues in the Gauss plane is preserved), but with the corresponding eigenfunction given by

(29) $$\vec{\rho }\left(y\right)={\vec{\rho }}_{0}\left(y\right)e^{-i\left(e/\hbar \right)\int_{0}^{\tilde{W}-\left|\tilde{W}-y\right|}A_{y}\left(\bar{x},\xi \right)\;d\xi }\;.$$

This means that

(30) $$\left\{\begin{array}{c} {\vec{\varphi }}^{\vec{K}}\left(y\right)={\vec{\varphi }}_{0}^{\vec{K}}\left(y\right)e^{-i\left(e/\hbar \right)\int_{0}^{y}A_{y}\left(\bar{x},\xi \right)\;d\xi }\\ {\vec{\varphi }}^{\vec{K^{\prime }}}\left(y\right)={\vec{\varphi }}_{0}^{\vec{K^{\prime }}}\left(y\right)e^{-i\left(e/\hbar \right)\int_{0}^{y}A_{y}\left(\bar{x},\xi \right)\;d\xi }\; \end{array}\right.$$

and thus

(31) $$\Phi_{\beta }^{\vec{\alpha }}\left(y\right)={\Phi_{\beta }^{\vec{\alpha }}}_{0}\left(y\right)e^{-i\left(e/\hbar \right)\int_{0}^{y}A_{y}\left(\bar{x},\xi \right)\;d\xi }$$

for $\vec{\alpha }=\vec{K},\vec{K^{\prime }}$ and β=A,B. Since $A_{y}\left(\bar{x},y\right)=B\bar{x}$, we have that $\Phi_{\beta }^{\vec{\alpha }}\left(y\right)={\Phi_{\beta }^{\vec{\alpha }}}_{0}\left(y\right)e^{-i\left(e/\hbar \right)B\bar{x}y}$. The exponential term represents the Peierls phase. Therefore, using this gauge, within each slice, it is sufficient to numerically solve the eigenproblem (14) for B=0. Then, in the following mode-matching procedure, the mode projections are calculated including the effect of the Peierls phases and operating on the Fourier coefficients of the functions ${\vec{\rho }}_{0}\left(y\right)$.

The adoption of this “second gauge” does not lead to ill-conditioned eigenproblems, and thus, in general, it is necessary for large values of the magnetic field and of the ribbon width.

Using this gauge, within each slice, we obtain the same number of propagating modes (i.e., of modes with $I_{x}\neq 0$) as in the absence of a magnetic field (because, as we have seen, the solutions with and without a magnetic field differ only for a phase factor). Then, the actual number of modes propagating through the device in the presence of a magnetic field (which in general differs from that in the absence of a magnetic field) is recovered in the simulation when a sufficient number of slices is composed. However, particular care has to be taken at the entrance and exit slices because the sum in the Landauer–Büttiker formula (Equation (22)) is performed only on the modes propagating in these slices. Therefore, using the “second gauge”, the final conductance result is limited by the number of modes propagating at the entrance and at the exit of the device in the absence of a magnetic field. This would lead to an underestimation of the conductance in the cases (which, as we will see, exist) in which the magnetic field actually leads to an increase of the conductance, if a proper model of the contacts is not used. Therefore, we have represented the entrance and exit contacts with regions (placed to the left of the first slice and to the right of the last slice), in which the potential energy is constant and exhibits a large step with respect to the inner region of the device (the actual sign of the potential step in the contacts is determined based on the nature of the carriers, which in turn depends on the position of the Fermi level with respect to the Dirac point: the potential is lower or higher in the contacts, with respect to the rest of the device, depending on whether transport is due to electrons or holes, respectively). These regions guarantee a number of modes injected into (and extracted from) the structure sufficiently larger than the number of modes that actually propagate in the device (which is a necessary requirement for the validity of the Landauer–Büttiker formula [63]). This approach matches the usual definition of input and output reservoirs, and it represents a good approximation for the band structure behavior at physisorbed contacts [13,64,65].

With this caution, the “second gauge” can be used in any situation, without numerical problems but, usually, with longer computational times. Instead, for low magnetic fields and narrow ribbons, when the problem is well conditioned, the “first gauge” is preferable because it allows faster simulations.

### 2.7. Generalization to the Case of a Space-Dependent Magnetic Field

In the general case in which the orthogonal magnetic field is not constant, but varies in space, i.e., $\vec{B}=B\left(x,y\right)\hat{z}$, the two gauges can be extended in such a way as to still satisfy the relationship $\vec{B}=\vec{\nabla }\times \vec{A}$.

The “first gauge” becomes $\vec{A}=\left[-\int_{0}^{y}B\left(x,\eta \right)d\eta \right]\;\hat{x}$. In this case, the slice length has to be chosen in such a way as to preserve within each slice the invariance along *x* of the ribbon structure, of *U*, and also of $\vec{A}$ (if $\vec{B}$, and thus also $\vec{A}$, varies with *x*). Inside each slice (centered on $\bar{x}$), the numerical procedure is analogous to that described in the case of a constant $\vec{B}$, with the difference that now, in general, $b\left(y\right)=-\left(e/\hbar \right)\int_{0}^{\tilde{W}-\left|\tilde{W}-y\right|}B\left(\bar{x},\eta \right)d\eta$ is not a triangular wave, but an even real function, the Fourier coefficients of which are not analytically known but, however, can be quickly computed with proper FFT routines.

Instead, the “second gauge” can be extended considering the vector potential $\vec{A}=\left[\int_{0}^{x}B\left(\eta ,y\right)d\eta \right]\;\hat{y}$. As in the case with constant $\vec{B}$, within each slice (chosen in such a way as to guarantee the independence on *x* of the ribbon structure, of *U*, and of $\vec{A}$), the calculation can be performed either solving the eigenproblem in the presence of the magnetic field or exploiting the Peierls phases. If we follow the first approach, we have to notice that now, in general, the function $c\left(y\right)=i\left(e/\hbar \right)\mathrm{sign}\left(\tilde{W}-y\right)\int_{0}^{\bar{x}}B(\eta ,\tilde{W}-|\tilde{W}-y\left|\right)d\eta$ is not a square wave but an odd imaginary function, the Fourier coefficients of which have to be computed with proper FFT calculations. Alternatively, we can adopt the approach with the Peierls phase factors, using the general form given by Equation (31) (since, in this case, B can depend on *y*).

## 3. Numerical Simulations

The simulation procedure that we have described has been implemented in a Fortran 77 code. The potential landscape is provided as an input, analytically or numerically (on a sufficiently detailed mesh). The code automatically partitions the device into a series of slices, with a maximum length chosen in such a way as to satisfy the constraints detailed in the previous section and to avoid the numerical problems that could otherwise derive from exponential terms. At the end of the calculation, the simulation code yields the value of the conductance (and, if needed, of other quantities that can be obtained from the scattering matrix of the device, and in particular from its transmission coefficients).

In order to test the validity of our method, we have applied it to the analysis of three situations characteristic of the behavior of graphene in the presence of a magnetic field: the energy-gap modulation that takes place in armchair graphene ribbons as the magnetic field is increased; the coherent electron focusing, which can be observed in properly designed structures before reaching the quantum Hall regime; and the Aharonov–Bohm effect appearing in the case of a circular *p*–*n* junction in the quantum Hall regime. The simulations have been performed assuming to operate with a small voltage difference between the contacts and at low temperature; this makes the energy averaging of the conductance superfluous, speeding up the calculations.

### 3.1. Energy-Gap Modulation

We have first simulated the transport properties of an armchair ribbon with width $\tilde{W}=150$ nm (corresponding to 1219 dimer lines) and length L=300 nm. The analysis of a structure with this size, much larger than in the numerical studies previously reported in the literature [66,67,68,69], has been made possible by the adoption of a continuum, less-computational demanding, model in the simulation code. We have computed the conductance *G* as a function of the Fermi energy *E* with which the electrons are injected into the ribbon, for different values of the orthogonal magnetic field B (0, 0.2, 0.4, 0.6, 0.8, and 1 T). These values of $\tilde{W}$ and B are sufficiently small that no ill-conditioning problem appears, also using the “first gauge”.

We have considered two configurations of the potential: a potential null over the whole device and a potential given by the sum of two Lorentzian functions with a 10 nm half-width at half-maximum (one with a −0.05 eV amplitude and centered on the point of the ribbon with x=100 nm, y=50 nm, and the other with a +0.05 eV amplitude and centered on the point with x=200 nm, y=100 nm).

#### 3.1.1. Simulations with Abrupt Contacts

**Figure 3:**
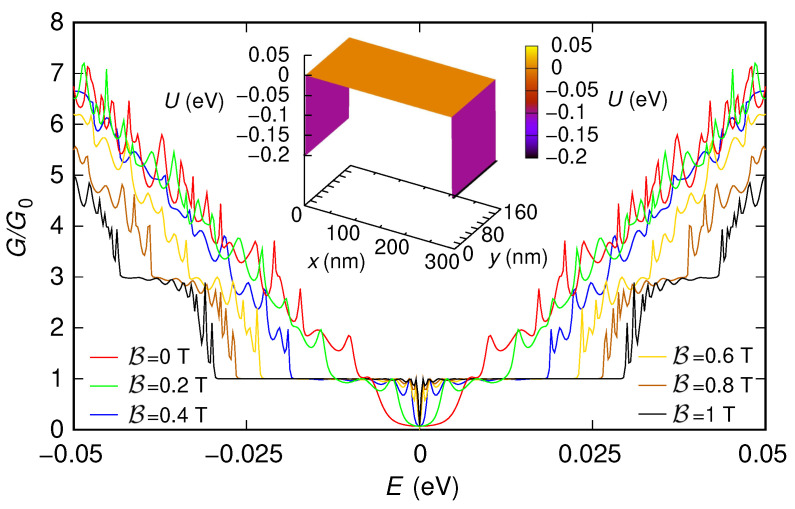
Conductance *G*, normalized with respect to the conductance quantum $G_{0}$, which was obtained as a function of the Fermi energy *E* for six values of the orthogonal magnetic field B for the 150 nm-wide and 300 nm-long graphene ribbon with zero potential and two 5 nm-long sections at the entrance and exit, which represent abrupt contacts (with a modulus of 0.2 eV for the potential steps of the contacts). In the inset, we represent the potential profile that we have considered.

**Figure 4:**
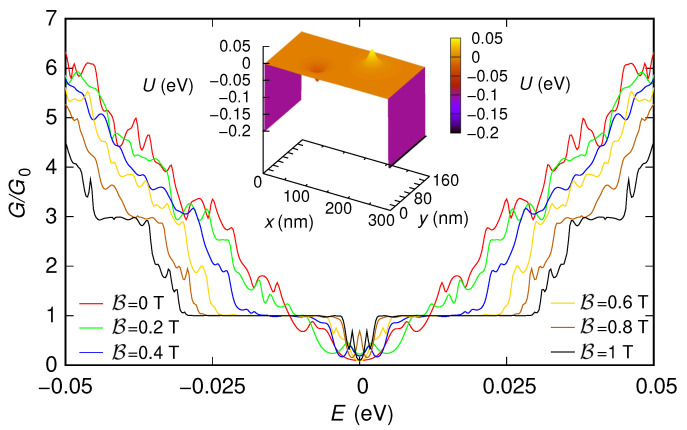
Conductance *G*, normalized with respect to the conductance quantum $G_{0}$, which was obtained as a function of the Fermi energy *E* for six values of the orthogonal magnetic field B for the 150 nm-wide and 300 nm-long graphene ribbon with a potential given by the sum of two Lorentzian functions and two 5 nm-long sections at the entrance and exit, which represent abrupt contacts (with a modulus of 0.2 eV for the potential steps of the contacts). In the inset, we represent the potential profile that we have considered.

First, we have included also two 5 nm-long sections (one at the entrance and the other at the exit of the device) characterized by a potential step with a large enough modulus (0.2 eV). As we have previously discussed, the presence of these sections, which emulate the effect of the contacts, is needed to use the “second gauge” without underestimating the conductance. In Figure 3 and Figure 4, we show the behavior of the conductance (normalized with respect to the conductance quantum $G_{0}=2e^{2}/h$) that we have obtained, as a function of the Fermi energy *E*, for the two configurations of the potential (reported in the insets of the two figures). We have verified that all the approaches we have discussed (i.e., using the “first gauge,” using the “second gauge” without Peierls phases, i.e., with a direct solution of the eigenproblem with B≠0 within each slice, and using the “second gauge” with the Peierls phase method) give the same results, even though with different simulation times. The technique with the “first gauge” (which requires a smaller number of slices) is the quickest one, followed by the method with the “second gauge” and Peierls phases (which requires a larger number of slices), and then by the approach with the “second gauge” but without Peierls phases (which, beyond requiring a large number of slices, involves the solution of a large-size eigenproblem in each slice). For example, in the case of the nonzero potential, the result for a magnetic field B=1 T and an injection energy (Fermi energy) E=0.05 eV have been obtained in 21 s, 1 min, and 7 min, respectively, on a processor Intel Core i7-4900MQ @ 2.80GHz (Intel, Santa Clara, CA, USA).

In the results shown in Figure 3 and Figure 4, we can notice several interesting features. For both potential configurations, the conductance around E=0 eV tends to increase as the magnetic field is increased. Moreover, in both cases, for large values of the magnetic field, a staircase behavior of the conductance as a function of the Fermi energy tends to appear, with a step height of two conductance quanta (except for the region around E=0).

#### 3.1.2. Simulations without Contacts

**Figure 5:**
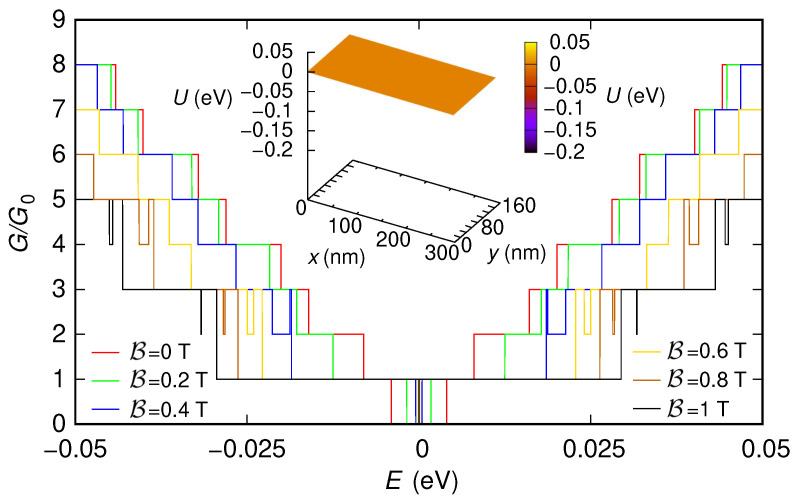
Conductance *G*, normalized with respect to the conductance quantum $G_{0}$, which was obtained as a function of the Fermi energy *E* for six values of the orthogonal magnetic field B for the 150 nm-wide and 300 nm-long graphene ribbon with zero potential and without including the contacts. These results have been obtained using the “first gauge”. In the inset, we represent the potential profile that we have considered.

**Figure 6:**
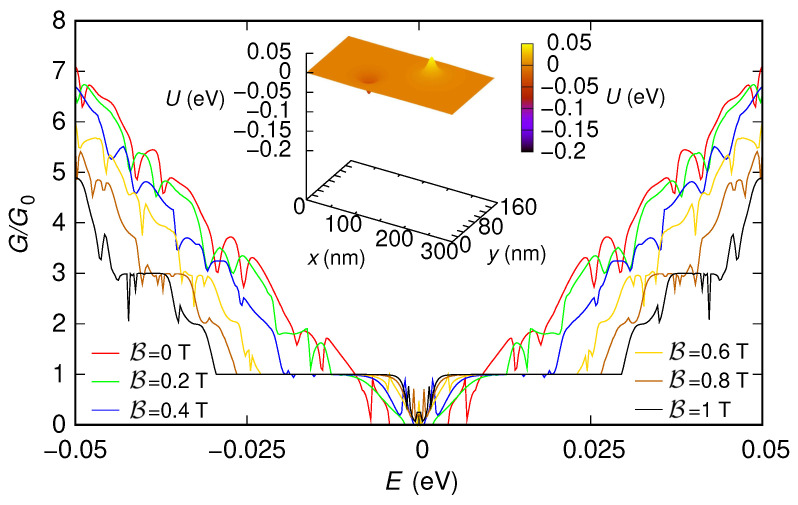
Conductance *G*, normalized with respect to the conductance quantum $G_{0}$, which was obtained as a function of the Fermi energy *E* for six values of the orthogonal magnetic field B for the 150 nm-wide and 300 nm-long graphene ribbon with a potential given by the sum of two Lorentzian functions and without including the contacts. These results have been obtained using the “first gauge”. In the inset, we represent the potential profile that we have considered.

These features, which in the presence of the two sections implementing the contacts are partially hidden by the tunneling effect between the contacts and by the reflections and interference effects produced by the abrupt transitions between the contacts and the actual device, appear more clearly if instead (in order to focus only on the behavior of the effective device) these contacts are not included. However, in this case, only the “first gauge” can be used, as we have previously discussed. In Figure 5 and Figure 6, we report the results obtained simulating, with the “first gauge,” the structure without the contacts: Figure 5 has been obtained for a potential vanishing everywhere, while Figure 6 has been obtained for the potential given by the sum of the two Lorentzian functions (the two potential profiles are reported in the insets of the two figures).

Let us first focus on the case of zero potential (Figure 5).

In this case, there is no scattering in the device; therefore, both in the absence and in the presence of a magnetic field, we observe a staircase behavior of the conductance as a function of the injection energy. Each new step corresponds to the activation of one or more transport modes, which are able to propagate across the device. The energy thresholds at which the steps start correspond to the extrema of the corresponding energy bands: starting from E=0, each time the injection energy, increasing (decreasing), crosses the minimum (maximum) of a conductance (valence) band, the corresponding transport mode begins to propagate through the device and to contribute to the conductance.

In the absence of a magnetic field, we notice the presence of an energy gap, i.e., the range of energy around E=0 for which the conductance is zero (while, in Figure 3, it was impossible to observe an energy gap due to the tunneling transmission between the contacts). Moreover, the height of each conductance step is equal to a quantum of conductance. This is due to the fact that the considered armchair ribbon, containing $N_{D}=1219$ dimer lines across its width, is semiconducting and thus exhibits an energy gap and no band degeneration in its dispersion relations [7].

Increasing the magnetic field, we observe that the energy gap is progressively reduced until it completely disappears (this effect has been experimentally observed in Ref. [70] and simulated with a tight-binding model in Refs. [66,67,68,69], where its exploitation for the fabrication of devices with large magnetoresistive modulation without ferromagnetic contacts was proposed). The staircase-shaped conductance behavior changes for a sufficiently high value of the magnetic field, with the conductance assuming only values equal to 2n+1 times the conductance quantum, with n≥0 an integer number. The reason of this behavior [66] is that, when the magnetic field is sufficiently high, transport takes place through edge states, propagating along the edges of the ribbon. The energy thresholds for the onset of these edge states are the Landau levels, which in monolayer graphene are doubly-degenerate (which justifies the fact that, in this case, the amplitude of each conductance step is equal to two conductance quanta). In particular, a Landau level is equal to 0 and is shared by electrons and holes [2]; therefore, around E=0, the conductance is nonzero and equal to a conductance quantum, without any energy gap. For intermediate values of the magnetic field, there is a transition between the situation for B=0 (with a nonzero energy gap) and that for sufficiently high B (with a zero energy gap). In particular, the lowest conduction band and the highest valence band tend to shift toward lower and higher energies, respectively, getting closer to each other and progressively reducing the energy gap until it finally disappears [66].

The presence of a nonzero potential alters these results (Figure 6). The change is clearly visible at low magnetic fields, where the staircase behavior of the conductance as a function of energy disappears as a consequence of the scattering action of the potential. However, for sufficiently high values of the magnetic field, the staircase behavior is substantially recovered. This is a consequence of the fact that, in this case, conduction takes place through edge states, which, propagating along the edges of the device, make the transport behavior substantially insensitive to the value of the potential in the ribbon.

**Figure 7:**
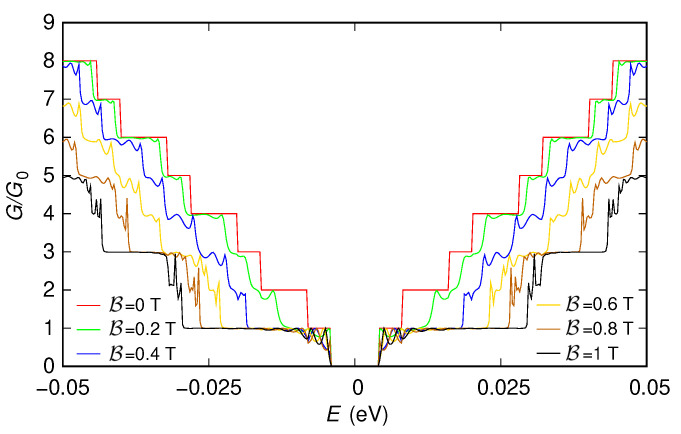
Conductance *G*, normalized with respect to the conductance quantum $G_{0}$, which was obtained as a function of the Fermi energy *E* for six values of the orthogonal magnetic field B for the 150 nm-wide and 300 nm-long graphene ribbon with zero potential and without including the contacts, using the “second gauge”. These results are not correct, since the absence of contacts limits the number of modes injected into the structure.

**Figure 8:**
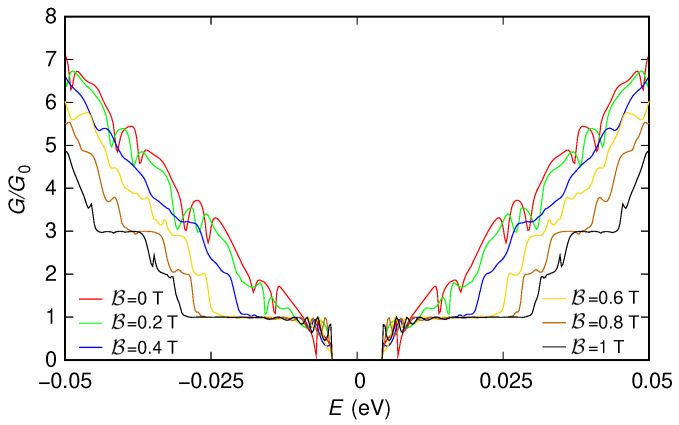
Conductance *G*, normalized with respect to the conductance quantum $G_{0}$, which was obtained as a function of the Fermi energy *E* for six values of the orthogonal magnetic field B for the 150 nm-wide and 300 nm-long graphene ribbon with a potential given by the sum of two Lorentzian functions and without including the contacts, using the “second gauge”. These results are not correct, since the absence of contacts limits the number of modes injected into the structure.

For comparison, in Figure 7 and Figure 8, we report (for the two potential profiles) the results obtained simulating the structure without contacts using the “second gauge” (either solving directly the eigenproblem with nonzero B or using the Peierls phases). As can be seen, the use of the “second gauge” for the structure without contacts gives wrong results. In particular, as we have previously discussed, the number of modes propagating through the device that is obtained using the “second gauge” can not be greater than the number of modes that propagate for B=0 in the input and output slices. Therefore, as confirmed by the reported results, the simulations with the “second gauge” of the structure without contacts are inherently unable to reproduce the decrease of the energy gap with an increasing magnetic field.

#### 3.1.3. Effect of Smooth Contacts

However, we remark that the same results that have been obtained with the “first gauge” on the structure without contacts can be achieved also (with either gauge) on the structure with the contacts if proper provisions are taken in order to minimize the effects of the contacts.

**Figure 9:**
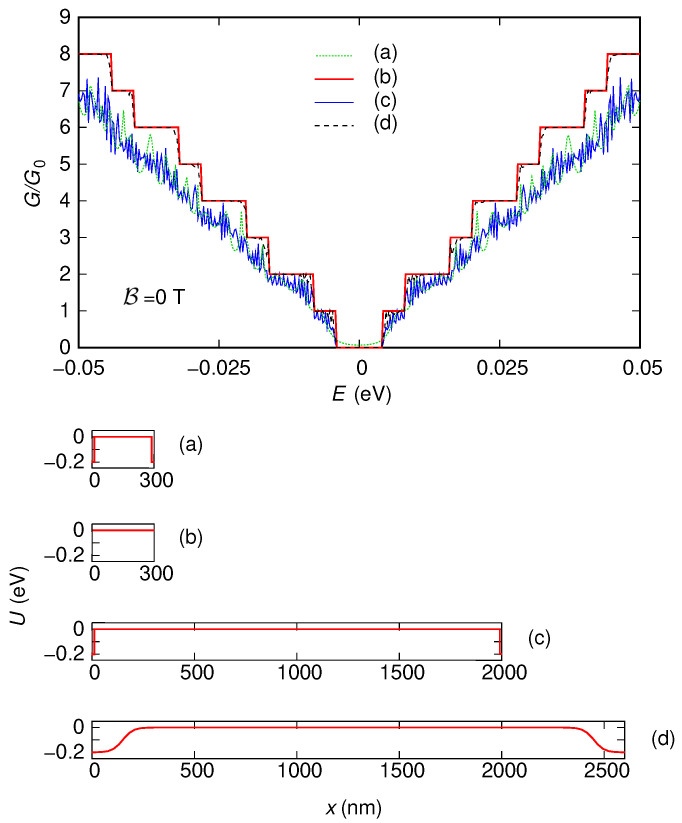
Conductance *G*, normalized with respect to the conductance quantum $G_{0}$, which was obtained as a function of the Fermi energy *E*, in the absence of an orthogonal magnetic field B and with a zero potential in the region of interest in the following four conditions: (**a**) 150 nm-wide and 300 nm-long graphene ribbon with 5 nm-long abrupt contacts at the entrance and at the exit; (**b**) 150 nm-wide and 300 nm-long graphene ribbon without including the contacts; (**c**) 150 nm-wide and 2000 nm-long graphene ribbon with 5 nm-long abrupt contacts at the entrance and at the exit; (**d**) 150 nm-wide and 2000 nm-long graphene ribbon with 300 nm-long smooth contacts at the entrance and at the exit. The longitudinal sections of the corresponding potential profiles are sketched in the lower part of the figure.

In detail, we have to significantly increase the length of the device (in such a way as to suppress the tunneling probability between the input and output contacts through the “barrier” represented by the device itself) and, at the same time, replace the abrupt transition between each contact and the device with a very smooth one (in such a way as to strongly reduce the reflections induced by these interfaces and the interference patterns that they generate). Proceeding in this way, we can use either of the considered gauges (since the presence of the contacts guarantees, already for B=0, a sufficiently high number of propagating modes in the input and output slices), but with much longer computational times (because much larger numbers of slices and of modes are needed). As an example, in Figure 9, we plot the results obtained for the ribbon with zero potential energy and in the absence of magnetic field. In detail, we report again the results obtained for the 150 nm-wide and 300 nm-long ribbon, without ((a) curve) and with ((b) curve) the contacts, represented with two 5 nm-long sections with a modulus of 0.2 eV for the abrupt potential steps of the contacts. Instead, with the curve (c), we report the conductance behavior obtained for a 150 nm-wide and 2000 nm-long ribbon with the same abrupt representation for the contacts. We notice that, as a consequence of the vanishing tunnel probability between the two contacts, the energy gap observed in the absence of the contacts is recovered; however, the rest of the behavior is very irregular, as a result of the reflections introduced by the abrupt contact–device interfaces and of the interference patterns they generate. Finally, the curve (d) represents the conductance behavior obtained for a 150 nm-wide and 2000 nm-long ribbon, preceded and followed by 300 nm-long regions, where the potential gradually varies from $U_{0}$ to 0 and vice versa (with $|U_{0}|=0.2$ eV) in this way: $U_{0}-\left(U_{0}/2\right)\left(1+\tanh\left(\left(x-150\;\mathrm{nm}\right)/\left(50\;\mathrm{nm}\right)\right)\right)$ and $U_{0}-\left(U_{0}/2\right)\left(1+\tanh\left(\left(-x+2450\;\mathrm{nm}\right)/\left(50\;\mathrm{nm}\right)\right)\right)$, respectively. The smoothness of these transitions mitigates the reflections and the interference effects deriving from the contact–device interfaces. As we can see, in this way, we achieve substantially the same behavior obtained without including the contacts ((a) curve).

### 3.2. Coherent Electron Focusing

**Figure 10:**
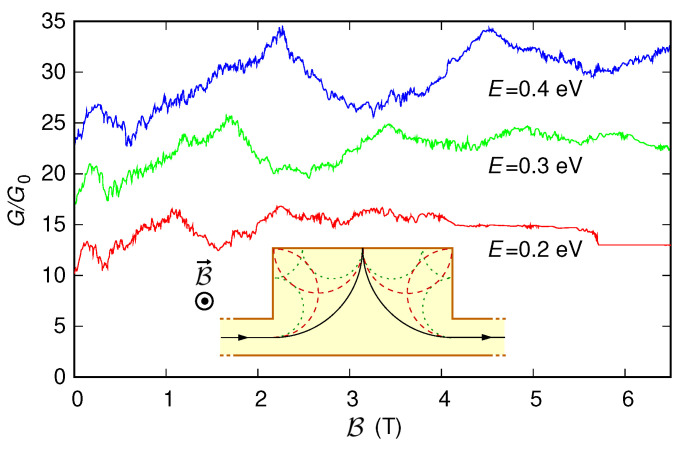
Conductance *G*, normalized with respect to the conductance quantum $G_{0}$, which was obtained as a function of the orthogonal magnetic field B for the structure represented in the inset, keeping the Fermi energy *E* constant at 0.2 eV (red line), 0.3 eV (green line), and 0.4 eV (blue line). In the inset, we represent also the three cyclotron trajectories that give rise to the first three conductance peaks.

Then, we have applied our code to the study of coherent electron focusing [43,71,72,73,74,75]. We have simulated the configuration sketched in the inset of Figure 10. In this case, the device consists of sections with different widths: the first and third sections are armchair ribbons with a width of 80 nm and a length of 450 nm, while the second section is an armchair ribbon with a width of 240 nm and a length of 400 nm. The three sections are aligned along the lower edge, while the geometry discontinuity takes place along the upper edge. The potential energy is zero everywhere, except for the first and last 300 nm-long sections. In these sections, the presence of the contacts is emulated with a potential starting with a modulus equal to 0.3 eV, which in the first 300 nm has a smooth transition toward zero with the hyperbolic-tangent-shaped behavior already adopted in the previous subsection; then, it has a constant zero value, and finally, it undergoes a smooth transition toward a modulus of 0.3 eV with an analogous hyperbolic-tangent-shaped behavior. We have evaluated the conductance through the structure as a function of the orthogonally-applied magnetic field, keeping the Fermi energy *E* constant at 0.2 eV (actually, for this value of the Fermi energy, the inclusion or not of the contacts does not appreciably affect the results). The simulations performed with all the approaches described in the method section give the same results, shown with a red line in Figure 10.

For low values of the magnetic field, we notice the presence of peaks in the conductance for values of the magnetic field that are multiples of a value $B^{*}$: $B^{*}=1.1445$ T, $2\;B^{*}=2.289$ T, and $3\;B^{*}=3.4335$ T. This behavior can be explained, as in Refs. [43,71], with the fact that for low values of the magnetic field, when a quantum Hall regime has not yet been reached, we can assume that the charges move along cyclotron orbits [75] with cyclotron radius [43,76]

(32) $$R_{c}=\frac{\left|p\right|}{eB}=\frac{\left|E\right|}{ev_{F}B}\;.$$

Since the length L=400 nm of the central section of the device is twice the distance (200 nm) between the center of the (entrance and exit) leads and the upper edge of the central section, when the electrons are injected from the entrance lead with a cyclotron radius equal to L/2, using a semi-classical description, they run along the path shown with a solid line in the inset of Figure 10 and arrive at the exit lead, with a single specular reflection at the middle of the upper edge of the central section. In this condition, a maximum of the conductance is expected. Enforcing $R_{c}=L/2$, we find that this actually happens for $B=2\;E/\left(ev_{F}L\right)=B^{*}=1.1445$ T. A similar explanation can be given for the conductance peaks observed for $2\;B^{*}=2.289$ T and $3\;B^{*}=3.4335$ T, since for these values of the magnetic field (and thus for the corresponding values of the cyclotron radius, which are equal to L/4 and L/6, respectively), the semi-classical electron trajectories are those represented with the dashed and dotted lines in the inset of Figure 10, respectively. The fluctuating conductance behavior superimposed with these main peaks derives from the complex scattering action of the boundaries, for all of which we have considered realistic boundary conditions rather than adopting absorbing boundary conditions as in Refs. [43,72]. These superimposed fluctuations would also be smeared down by energy averaging if the simulations were performed at higher temperature [71]. Further increasing the value of the magnetic field, we see that, after a crossover region with an intermediate behavior, a quantum Hall regime is reached, with an integer number of edge states transmitted nearly perfectly and thus a conductance value multiple of the conductance quantum. As a rule of thumb, this regime is achieved when the cyclotron diameter becomes sufficiently smaller than the width $W_{lead}$ of the entrance and exit sections, which in this case means for a value of the magnetic field B sufficiently higher than 5.7226 T (the value of B for which $2R_{c}=W_{lead}=80$ nm).

In Figure 10, we report also, with a green and a blue line, the behavior of the conductance as a function of the magnetic field obtained in the same device keeping the Fermi energy *E* constant at 0.3 eV and 0.4 eV, respectively. These curves confirm the previous discussion, exhibiting peaks at $B^{*}=2\;E/\left(ev_{F}L\right)=1.717$ T, $2\;B^{*}=3.434$ T, and $3\;B^{*}=5.151$ T in the case of E=0.3 eV, and at $B^{*}=2\;E/\left(ev_{F}L\right)=2.289$ T and $2\;B^{*}=4.578$ T in the case of E=0.4 eV.

We remark that in contrast to previously reported numerical studies, in which tight-binding or semi-analytical continuum solutions were adopted, our code can in principle handle large structures with a generical potential (including, for example, the electrostatic effect of a scanning tip considered in Refs. [74,75]).

### 3.3. Aharonov–Bohm Interferometer

#### 3.3.1. Simulation of a Narrow Structure

Then, in order to further test the code, we have simulated the structure described in Ref. [30]. The device consists of a graphene armchair ribbon in which a negatively charged floating tip, placed over the point $\left(x_{0},y_{0}\right)$ of the ribbon, induces a Lorentzian-shaped potential landscape of the form $U\left(x,y\right)=U_{0}/\left(1+\left[{\left(x-x_{0}\right)}^{2}+{\left(y-y_{0}\right)}^{2}\right]/d^{2}\right)$, with $U_{0}$ the maximum and *d* the half-width at half-maximum of the potential. The charge carriers are injected into the device with a Fermi energy *E* such that $0<E<U_{0}$. The circumference (with radius $R=d\sqrt{(U_{0}/E)-1}$) corresponding to the points for which U(x,y)=E separates an *n*-type region (the part of the graphene ribbon outside the circumference) from a *p*-type region (the part inside the circumference) and thus represents the interface of a circular *p*–*n* junction [30,77]. The point $\left(x_{0},y_{0}\right)$ is taken at the center of the ribbon and the values *E*, $U_{0}$, and *d* in such a way that the circumference is completely within the ribbon but quite close to the ribbon edges. In this condition, when the magnetic field is large enough and the quantum Hall regime is reached, a part of the current flowing near the ribbon edge can couple to the *p*–*n* junction and circulate around it. If the ribbon is semiconducting, the regions in which the circular *p*–*n* junction and the ribbon edges approach act as beam splitters [78], and the current flow splits into different paths around the *p*–*n* junction. The interference between the different current paths gives rise to the Aharonov–Bohm effect, which manifests itself through oscillations [30] in the behavior of the transmission as a function of the magnetic field, with period equal to ΔB=h/(eA), where $A=\pi R^{2}$ is the area of the region enclosed by the circumference.

**Figure 11:**
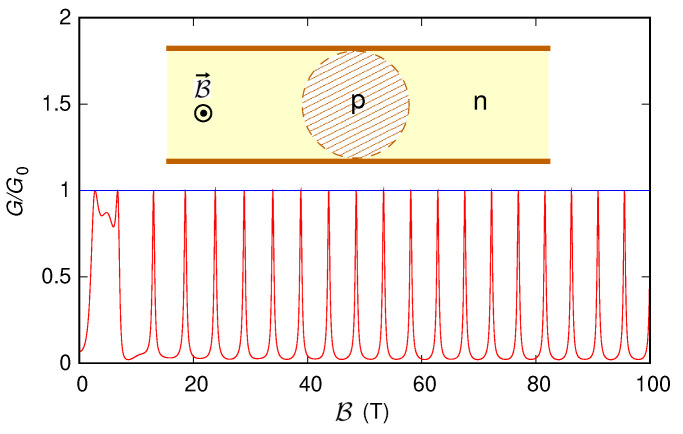
Conductance *G*, normalized with respect to the conductance quantum $G_{0}$, which was obtained as a function of the orthogonal magnetic field B for the ribbon (with length 120 nm and width ∼36 nm) represented in the inset, where the presence of a Lorentzian-shaped potential introduces a circular *p*–*n* junction. The red curve indicates a semiconducting ribbon, while the blue line indicates a metallic one.

We have applied our approach to the structures that had been studied (using a tight-binding-based quantum transmitting boundary method) in Ref. [30]. In detail, we have considered a 120 nm-long semiconducting ribbon with an effective width $\tilde{W}=36.032$ nm (corresponding to 292 dimer lines of carbon atoms between the edges, i.e., to W=35.79 nm), a Fermi energy E=30 meV, and a Lorentzian potential energy with $U_{0}=400$ meV, d=4.92 nm, and $\left(x_{0},y_{0}\right)$ at the center of the ribbon ($x_{0}=60$ nm and $y_{0}=18.016$ nm, if we take x=0 at the beginning of the actual 120 nm-long ribbon and y=0 at its lower effective edge). In this case, the circumference radius is R=17.27 nm (inset of Figure 11), and the corresponding theoretical period of the Aharonov–Bohm oscillations is ΔB=4.41 T. We have achieved approximately the same results both using the “first gauge” without including the presence of the contacts and using either of the two gauges with smooth contacts. In Figure 11, we report with a red line the behavior that we have obtained for the conductance as a function of the magnetic field. These results are very similar to those obtained in Ref. [30] with a tight-binding model; in particular, for B>10 T (i.e., in the quantum Hall regime), we observe periodic oscillations with a period of about 4.7 T, which is in quite good agreement with the theoretically predicted value. Instead, repeating the simulation for a metallic ribbon with 293 dimer lines of carbon atoms across its width (and thus with W=35.91 nm and $\tilde{W}=36.155$ nm), with the probe placed over the center of the ribbon, i.e., over the point $\left(x_{0},y_{0}\right)$ with $x_{0}=60$ nm and $y_{0}=18.077$ nm, we have obtained the behavior represented in Figure 11 with the blue line. Indeed, as shown in Refs. [30,78], in the case of metallic ribbons, in the regions where a ribbon edge and the circular *p*–*n* junction approach, the edge current is able to transfer completely between the edge and the junction, without splitting, and, using an envelope-function approximation, no Aharonov–Bohm oscillation appears.

#### 3.3.2. Simulation of a Wider Structure

**Figure 12:**
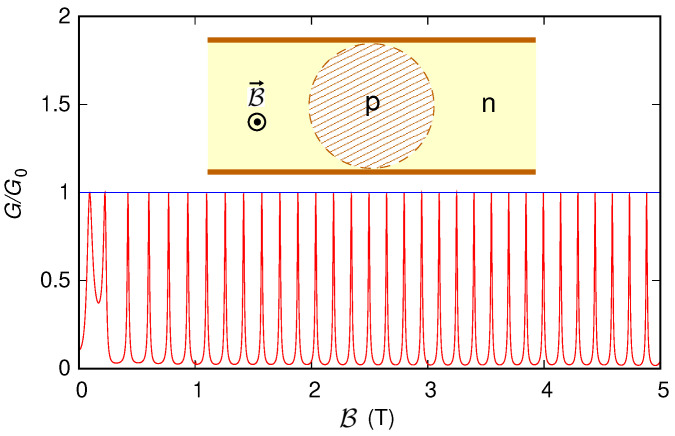
Conductance *G*, normalized with respect to the conductance quantum $G_{0}$, which was obtained as a function of the orthogonal magnetic field B for the ribbon (with length 500 nm and width ∼200 nm) represented in the inset, where the presence of a Lorentzian-shaped potential introduces a circular *p*–*n* junction. The red curve indicates a semiconducting ribbon, while the blue line indicates a metallic one.

Then, exploiting the advantage of the envelope-function approach in terms of lower computational requirements and shorter simulation times, we have analyzed a larger (and more realistic, also in terms of the magnitude of the applied magnetic field) semiconducting ribbon with a length of 500 nm and an effective width $\tilde{W}=200.2$ nm (corresponding to 1627 dimer lines of carbon atoms between the edges and to W=199.96 nm). In this case, we have considered a Fermi energy E=5.5 meV and a Lorentzian potential energy with $U_{0}=70$ meV, d=28 nm, and $\left(x_{0},y_{0}\right)$ at the center of the ribbon ($x_{0}=250$ nm and $y_{0}=100.1$ nm, measured from the beginning of the actual 500 nm-long ribbon and from its lower effective edge). With these values, the radius of the circumference is R=95.89 nm (inset of Figure 12), and the theoretically predicted period of the Aharonov–Bohm oscillations is ΔB=0.143 T. The results are reported in Figure 12 with a red line; also in this case, we notice that, for B>0.3 T (i.e., in the quantum Hall regime), the conductance oscillates with a period of 0.148 T, which is close to the theoretical value. Repeating the simulation for a metallic ribbon with 1628 dimer lines of carbon atoms across its width (W=200.08 nm, $\tilde{W}=200.327$ nm), with the probe placed over the ribbon center, $\left(x_{0},y_{0}\right)$ with $x_{0}=250$ nm and $y_{0}=100.16$ nm, we have obtained the behavior represented in Figure 12 with the blue line, without, again, any visible Aharonov–Bohm oscillation. This numerical validation for more realistic device sizes and magnetic field values represents a step ahead in view of a future experimental verification of this phenomenon.

## 4. Conclusions

We have presented an envelope-function based method for the simulation of transport in graphene in the presence of an orthogonal magnetic field and of a generic electrostatic potential. Our method has the advantage, with respect to more accurate atomistic approaches, of significantly reducing the computational effort, and therefore allowing us to treat graphene structures with a size large enough to be of practical interest.

We have considered a few alternative approaches for the introduction of magnetic field, differing in terms of the choice of gauge for the vector potential and in terms of the specific numerical procedure used in the calculation. We have assessed their range of applicability and compared their performance in terms of computational times. In particular, we have shown that the possibility to study the problem using different gauges makes it possible to overcome the ill-conditioning issues from which some of these techniques may be affected in particularly challenging scenarios.

Moreover, we have discussed some subtle aspects, such as the influence of the contact models, that require particular care in the simulations. We remark that many of these considerations can be useful also for the analysis of devices that are not specifically based on graphene.

With our code, we have been able to successfully reproduce several effects resulting from an orthogonal magnetic field in graphene-based devices (energy-gap modulation, coherent electron focusing, Aharonov–Bohm interference), which span from the semi-classical regime of cyclotron trajectories to the quantum Hall regime of edge state propagation. In particular, we have obtained results in agreement with those of the experimental and theoretical studies reported in the literature. Exploiting the computational efficiency of our code, we have been able to perform an analysis for many values of the magnetic field and of the injection energy on structures with sizes up to hundreds of nanometers and with a variety of potential landscapes. This has enabled us to confirm the interpretation of the reported physical phenomena and experimental observations on an extended parameter range.

More in general, our approach can be useful (self-consistently coupled with a proper electrostatic solver) for the simulation of transport in graphene-based devices with a generic gate configuration and an applied orthogonal magnetic field, especially for device sizes (often appearing in the applications, such as in the case of sensors) that are too large for an effective application of resource-demanding atomistic-level simulations.

## Data Availability

The numerical data used to support the findings of this study are available from the corresponding author upon request.
